# Cement for Rubber

**Published:** 1881-04

**Authors:** 


### CEMENT FOR RUBBER.

Powdered shellac is softened in ten times its weight of strong water of ammonia, whereby a transparent mass is obtained, which becomes fluid after keeping some little time without the use of hot water. In three or four weeks the mixture is perfectly liquid, and, when applied, it will be found to soften the rubber. As soon as the ammonia evaporates the rubber hardens again—it is said quite firmly—and thus becomes impervious both to gases and to liquids. For cementing sheet rubber, or rubber material in any shape, to metal, glass, and other smooth surfaces, the cement is highiy recommended.—*Scientific American.*

